# Neonatal and early‐onset diabetes in Ukraine: Atypical features and mortality

**DOI:** 10.1111/dme.15013

**Published:** 2022-12-15

**Authors:** Evgenia Globa, Nataliya Zelinska, Matthew B. Johnson, Sarah E. Flanagan, Elisa De Franco

**Affiliations:** ^1^ Ukrainian Scientific and Practical Center of Endocrine Surgery Transplantation of Endocrine Organs and Tissues of the Ministry of Health of Ukraine Kyiv Ukraine; ^2^ Institute of Biomedical and Clinical Science, Faculty of Health and Life Sciences University of Exeter Exeter UK

**Keywords:** mortality, neonatal diabetes, rare cases

## Abstract

**Aims:**

The aim of this study is to elucidate the aetiology and clinical features of neonatal and early‐onset diabetes in a large database for pediatric diabetes patients in Ukraine.

**Methods:**

We established a Pediatric Diabetes Register to identify patients diagnosed with diabetes before 9 months of age. Genetic testing was undertaken for 66 patients from 65 unrelated families with diabetes diagnosed within the first 6 months of life (neonatal diabetes, *n* = 36) or between 6 and 9 months (early‐onset diabetes, *n* = 30).

**Results:**

We determined the genetic aetiology in 86.1% of patients (31/36) diagnosed before 6 months and in 20% (6/30) diagnosed between 6 and 9 months. Fourteen individuals (37.8% of those with a genetic cause identified) had activating heterozygous variants in *ABCC8* or *KCNJ11*. An additional 10 individuals had pathogenic variants in the *INS* or *GCK* genes, while 4 had 6q24 transient neonatal diabetes. Rare genetic subtypes (including pathogenic variants in *EIF2AK3, GLIS3, INSR, PDX1, LRBA, RFX6* and *FOXP3)* were identified in nine probands (24.3% of solved cases), 6 of whom died. In total, eight individuals died between infancy and childhood, all of them were diagnosed before 6 months and had received a genetic diagnosis.

**Conclusions:**

In the last decade, the increased availability of comprehensive genetic testing has resulted in increased recognition of the contribution of rare genetic subtypes within pediatric diabetes cohorts. In our study, we identified a high mortality rate among these patients.

**“What is already known?”**

Neonatal diabetes mellitus is defined as diabetes diagnosed in the first 6 months of life, with some cases presenting between 6 and 9 months of age (early‐onset diabetes). While cohort studies in Europe, Japan and the United States have reported *de novo* variants in *KCNJ11* as the most common cause of permanent neonatal diabetes mellitus (PNDM), in countries with high rate of consanguineous marriages the inheritance pattern of PNDM is very different, with autosomal recessive causes being most common.

**“What this study has found?”**

Our data show a high proportion of rare genetic causes, including autosomal recessive aetiologies, within our cohort (24.4%), and a high mortality rate (21.6% of individuals with a confirmed genetic diagnosis). To our knowledge, this is the first study assessing mortality in a large neonatal/early‐onset diabetes cohort in a European country.

Clinical follow‐up in our cohort highlighted atypical presentation and clinical course of diabetes in some families.

**“What are the implications of the study?”**

Implementation of early comprehensive genetic testing including targeted next‐generation sequencing can improve clinical management of this genetically heterogeneous disease.

## 1 INTRODUCTION

Neonatal diabetes is defined as diabetes diagnosed in the first 6 months of life. It is a rare genetic disease affecting 1 in 90,000 to 160,000 live births.^1^ Rarely, some individuals can present with diabetes between 6 and 9 months of age (early‐onset diabetes); however, the vast majority of individuals diagnosed in this age group do not have a genetic cause for their disease and are most likely to have type 1 diabetes. Pathogenic activating variants in the genes encoding the ATP‐sensitive potassium channel (KATP) subunits (*KCNJ11* and *ABCC8*), dominant variants in the *INS* gene and chromosome 6q24 methylation abnormalities are the most common causes of neonatal diabetes in European countries.^2^, ^3^, ^4^, ^5^ Pathogenic variants affecting over 25 additional genes cause rarer subtypes of the disease,^6^ with 1–2 novel causes being reported each year.

The genetic causes of neonatal and early‐onset diabetes vary depending on geography, ancestry and consanguinity. While cohort studies in Europe, Japan and the United States have reported *de novo* variants in *KCNJ11* as the most common cause of permanent neonatal diabetes mellitus (PNDM),^4^, ^7^, ^8^, ^9^, ^10^, ^11^, ^12^ in countries with a high rate of consanguineous marriages, the inheritance pattern of PNDM is very different. In these settings, autosomal recessive subtypes are more common, with homozygous *EIF2AK3* variants causing Wolcott–Rallison syndrome being the most common cause of PNDM.^13^, ^14^ Wolcott–Rallison syndrome is a syndromic form of neonatal/early‐onset diabetes associated with a poor prognosis and high mortality rate in infancy.^15^ Early mortality in patients with neonatal/early‐onset diabetes has been reported in individuals with other rare genetic subtypes, such as those with monogenic autoimmune diabetes (including IPEX syndrome and diabetes caused by recessive *LRBA* variants)^16^, ^17^ and Donohue syndrome.^18^ The contribution of rare genetic subtypes, including those discovered in the last 5 years, in European countries is not clear, as is the disease's mortality rate.

We previously reported the genetic causes in 42 cases of neonatal and early‐onset diabetes occurring within the first 9 months of life in Ukraine and investigated treatment change in patients with *KCNJ11* or *ABCC8* pathogenic variants.^19^ Following on from this study, we now report 24 additional patients and focus our investigation on rare genetic causes diagnosed in recent years, atypical features and mortality rate.

## 2 METHODS

### 2.1 Subjects

A neonatal and early‐onset diabetes section of the Ukrainian Pediatric Diabetes Registry (UPDR) was created in 2012 to include individuals diagnosed with diabetes before 9 months of age identified by regional Ukrainian pediatric endocrinologists. Annual meetings on reconciliation of UPDR with regional endocrinologists were held by the Ukrainian authors of the article in accordance with the order of the Ministry of Health of Ukraine. All patients received insulin therapy free of charge according to the UPDR, so all patients with type 1 diabetes were included and the coverage was 100%. The Registry also contains data on patients with type 2 and monogenic diabetes.^20^ We present data from the UPDR starting from Jan 2013 to Dec 2021.

GAD and IA2 antibody testing was performed in individuals diagnosed between 6 and 9 months. The 30 individuals who were negative for both were further investigated through genetic testing.

### 2.2 Genetic testing

Genetic testing was undertaken using a combination of Sanger sequencing, targeted next‐generation‐sequencing (tNGS) for all known neonatal diabetes genes (full list available on https://www.diabetesgenes.org) and methylation analysis for chromosome 6q24 abnormalities as previously described.^13^ A type 1 diabetes (T1D) genetic risk score (T1D GRS) was calculated by next‐generation sequencing of 30 T1D‐associated SNPs.^21^ Patient 38 was tested by the Invitae laboratory (USA) using their tNGS Monogenic Diabetes Panel.

### 2.3 Clinical features

Clinical features at diagnosis and subsequent follow‐up were collected from all patients. For 42 individuals who were previously reported, we collected follow‐up information from hospital records and follow‐up visits.

### 2.4 Statistical analysis

Clinical characteristics are presented as median (interquartile range). For quantitative data Kruskal–Wallis test was used for comparative statistics.

## 3 RESULTS

We identified 70 cases with diabetes diagnosed before 9 months of age from the UPDR. DNA samples were available for 66 individuals from 65 unrelated families (94.2%). When available, variant testing was performed in family members by Sanger sequencing (primers available on request). The initial presentation for 42 of these individuals has been previously described.^19^

Thirty‐six individuals within our cohort were diagnosed before 6 months and therefore had neonatal diabetes. Diabetes remitted in 10 of them (27.8%), resulting in a diagnosis of transient neonatal diabetes. Thirty individuals had antibody‐negative, early‐onset diabetes diagnosed between 6 and 9 months. Summary of clinical characteristics for the two groups are presented in Table 1.

**TABLE 1:** Clinical characteristics of diabetes in 0–6 and 6–9 months groups

	Sex (f/m), %	Age of onset of diabetes, days	Birth weight, g	Gestation, weeks	BGL, mmol/L, at presentation	DDI, U/kg, at presentation	C‐peptide, ng/ml, at presentation	HbA_1c_, %, at presentation	Mortality (%)
PNDM, onset before 6 months, *n* = 26	67.8/32.2	100 [67.7;160.5]	2800 [2500;3095]	39 [37.6;40]	21 [8.4;26.5]	0.7 [0.1;1]	0.2 [0.07;0.5]	7.5 [6.2;9.6]	23%
TNDM, onset before 6 months, *n* = 10	60/40	20 [4;76.2]	2275 [2002;2772]	37.3 [34.6;39]	20.7 [17.6;26.5]	0.5 [0.25;1.4]	0.8 [0.6;1.2]	5.4 [4.3;6.4]	30%
Onset between 6 and 9 months, *n* = 30	42.9/57.1	240 [207.7;261]	3250 [3042;3500]	40 [39;40]	24,5 [22,6; 28,3]	0.6 [0.5;0.85]	0.05 [0.04;0.17]	8.8 [7.9;10.0]	0%
p value		<0.01	<0.01	<0.05			<0.01	<0.01	

**FIGURE 1:**
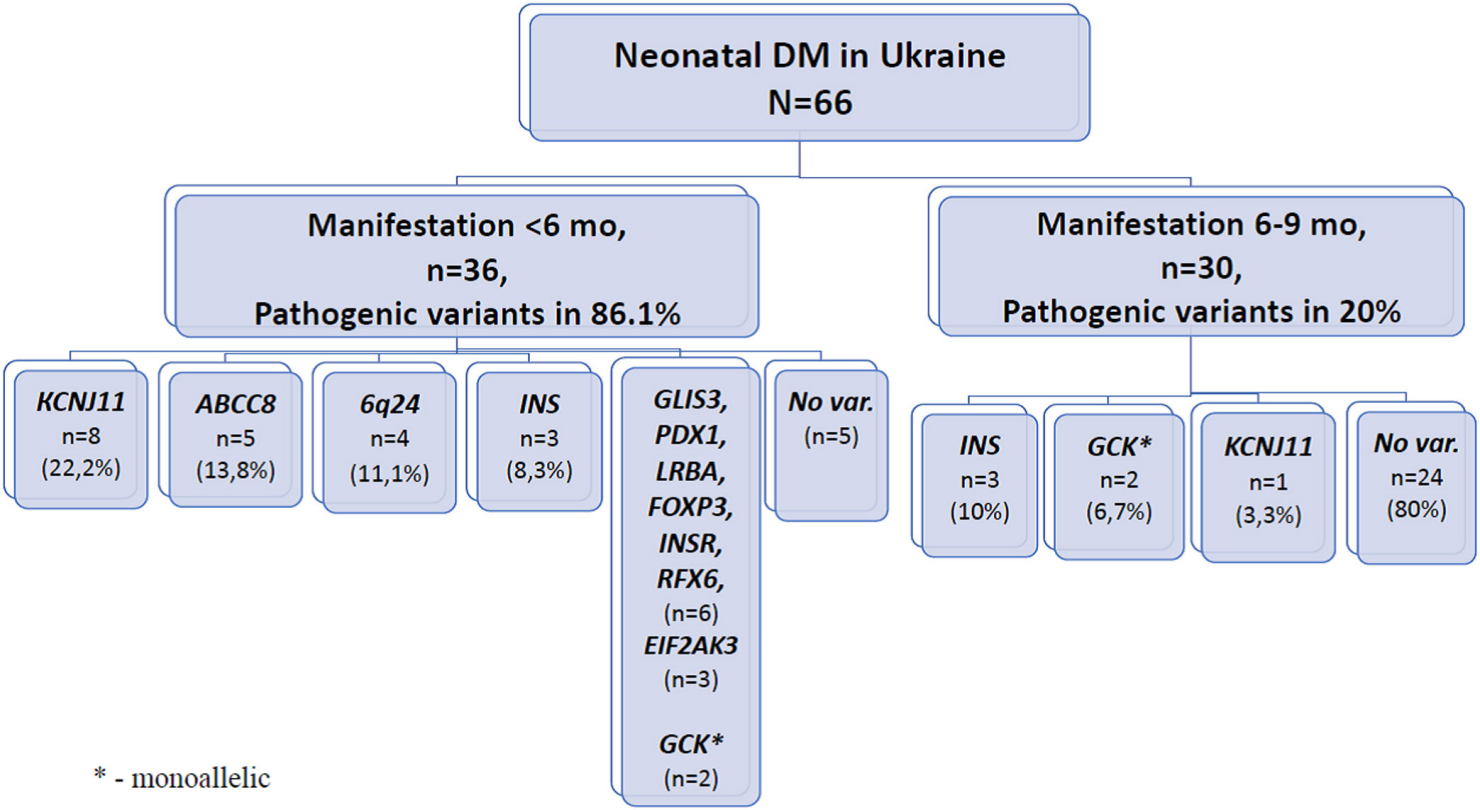
The distribution of neonatal (<6 months) and early‐onset (6–9 months) diabetes in Ukraine according to age at diagnosis and genetic aetiology.

Disease‐causing variants were identified in 37 patients. This included 31/36 (86.1%) individuals diagnosed before 6 months (neonatal diabetes) and 6/30 (20%) individuals diagnosed between 6 and 9 months (early‐onset diabetes) (Figure 1 and Table 2). Fourteen probands (37.8% of solved cases) harbored pathogenic activating variants in one of the KATP channel genes, *ABCC8* or *KCNJ11*. Dominant variants in the *INS* and *GCK* genes were identified in six and four individuals, respectively. Methylation abnormalities at 6q24 were identified in four individuals. Causative variants in the rarer causative genes (*EIF2AK3, GLIS3, INSR, PDX1, LRBA, RFX6* and *FOXP3*) were found in a total of nine individuals (24.4% of solved cases), six of whom (66.7%) died in early infancy/childhood.

**TABLE 2:** Genetic causes of neonatal and early‐onset diabetes mellitus in patients from the Ukrainian Pediatric Diabetes Registry

Patient	Clinical diagnosis	Age at diagnosis	Gene	Variant protein description	DNA description	References	Zygosity	Inheritance	ClinVar (Variation ID)	Pathogenicity (ACMG)
1	PNDM	3 months	*KCNJ11*	p.(Arg201Cys)	c.601C > T	^19^	Heterozygous	*de novo*	8668	Pathogenic
2	PNDM	2 months	*KCNJ11*	p.(Arg201Cys)	c.601C > T	^19^	Heterozygous	*de novo*	8668	Pathogenic
3	PNDM	4 months	*KCNJ11*	p.(Arg201Cys)	c.601C > T	^19^	Heterozygous	*de novo*	8668	Pathogenic
4	PNDM, iDEND	1 month	*KCNJ11*	p.(Val59Met)	c.175G > A	^19^	Heterozygous	*de novo*	8667	Pathogenic
5	PNDM, iDEND	2 months	*KCNJ11*	p.(Val59Met)	c.175G > A		Heterozygous	*de novo*	8667	Pathogenic
6	PNDM	1st day	*KCNJ11*	p.(Arg201His)	c.602G > A	^19^	Heterozygous	affected M at 3 months	8666	Pathogenic
7	PNDM	7 months	*KCNJ11*	p.(Gly53Asp)	c.158G > A	^19^	Heterozygous	*de novo*	8685	Pathogenic
8	TNDM	5 months	*KCNJ11*	p.(Glu229Lys)	c.685G > A	^19^	Heterozygous	*de novo*	158,683	Pathogenic
9	PNDM	1st day	*KCNJ11*	p.(His46Tyr)	c.136C > T		Heterozygous	affected M at 3.5 months	NA	Pathogenic
10	TNDM, DEND	3 months, died at 5 y.o.	*ABCC8*	p.(Ile49Phe)	c.145A > T	^19^	Heterozygous	*de novo*	NA	Pathogenic
11 Sibling of patient 10	TNDM, DEND	6 months, died at 9 y.o.	*ABCC8*	p.(Ile49Phe)	c.145A > T	^19^	Heterozygous	*de novo*	NA	Pathogenic
12	PNDM, DEND	2 months	*ABCC8*	p.(Val324Met)/p.(Arg1394Leu)	c.970G > A/c.4181G > T	^19^	Compound Heterozygous	Unaffected parents	1,338,342/NA	Pathogenic/ Pathogenic
13	TNDM	4 days	*ABCC8*	p.(Ile585Thr)	c.1754 T > C	^19^	Heterozygous	affected M at 21 y.o.	NA	Likely pathogenic
14	TNDM	1st day	*ABCC8*	p.(Arg826Trp)	c.2476C > T		Heterozygous	affected M at 26 y.o.	1,338,472	Pathogenic
15	PNDM	8 months	*INS*	p.(Gly32Ser)	c.94G > A	^19^	Heterozygous	*de novo*	21,122	Pathogenic
16	NDM with relapse	4 months	*INS*	p.(Gly32Ser)	c.94G > A		Heterozygous	affected M at 3 y.o.	21,122	Pathogenic
17	NDM with relapse	7 months	*INS*	p.(Gly32Ser)	c.94G > A	^19^	Heterozygous	Mosaic unaffected F	21,122	Pathogenic
18	PNDM	8 months	*INS*	p.(Leu41Pro)	c.122 T > C	^19^	Heterozygous	*de novo*	NA	Likely pathogenic
19	PNDM	4 months	*INS*	p.(Cys96Tyr)	c.287G > A	^19^	Heterozygous	*de novo*	13,387	Pathogenic
20	PNDM	5 months	*INS*	p.(Arg89Cys)	c.265C > T		Heterozygous	*de novo*	21,117	Pathogenic
21	TNDM with relapse at 10 y.o.	1 month	6q24			^19^	UPD	UPD		
22	TNDM with relapse at 9 y.o.	10 days	6q24			^19^	UPD	UPD		
23	TNDM	2 days	6q24			^19^	UPD	UPD		
24	TNDM	4 days	6q24				UPD	UPD		
25	PNDM	3 months	*EIF2AK3*	p.(Glu419fs)/ p.(Gly1010Val)	c.1254_1257delins26 /c.3029G > T	^19^	Compound heterozygous	Unaffected parents (carriers)	NA/NA	Pathogenic/Likely pathogenic
26	PNDM	2 months, died at 26 months	*EIF2AK3*	p.(Gly1010Val)	c.3029G > T	^19^	Homozygous	Unaffected parents (carriers)	NA	Likely pathogenic
27	PNDM	3 months, died at 3 months	*EIF2AK3*	p.(Asp164fs)/p.(Glu421fs)	c.492_495del/c.1254_1257 delins26		Compound heterozygous	Unaffected parents (carriers)	1,453,040/NA	Pathogenic/ Pathogenic
28	IFG	5 months	*GCK*	p.(Glu395Ter)	c.1183G > T	19, 22	Heterozygous	affected M	NA	Pathogenic
29	IFG	8 months	*GCK*	p.(Gly246Ala)	c.737G > C		Heterozygous	affected M	NA	Pathogenic
30	IFG	3 months	*GCK*	p.(Ile225Thr)	c.674 T > C		Heterozygous	affected M	NA	Likely pathogenic
31	IFG	8 months	*GCK*	p.(Thr228Ala)	c.682A > G		Heterozygous	affected M	447,413	Likely pathogenic
32	PNDM	7 days	*GLIS3*	p.(Pro444fs)/p.(His647Arg)	c.na/c.na	^19^	Heterozygous	Unaffected parents (carriers)	NA/NA	Pathogenic/Likely pathogenic
33	PNDM	1 month, died at 3 months	*FOXP3*	p.(Arg347His)	c.1040G > A		Hemizygous	Unaffected M (carrier)	647,073	Pathogenic
34	PNDM	5 months, died at 9 months	*LRBA*	p.(Glu946Ter)	c.2836_2839del		Homozygous	Unaffected parents (carriers)	1,458,881	Pathogenic
35	TNDM	1 month, died at 4 months	*INSR*	p.(Tyr94Ter)/p.(Arg1020Ter)	c.282 T > G/ c.3058C > T		Compound heterozygous	Unaffected M (carrier)/*de novo*	NA/NA	Pathogenic/Pathogenic
36	PNDM	17 days, died at 6 months	*PDX1*	p.?	c.1A > G		Homozygous	Unaffected parents (carriers)	NA	Likely pathogenic
37	PNDM	2nd day	*RFX6*	p.(Met409Thr)/p.(Ser517Arg)	c.1226 T > C/ c.1551 T > G		Compound heterozygous	Unaffected parents (carriers)	NA/NA	Likely pathogenic/Likely pathogenic

Abbreviations: F, father; IFG, impaired fasting glucose; M, mother; NA, not available.

In total 8 individuals in our cohort died between the ages of 3 months and 9 years. All of them were diagnosed before the age of 6 months and had a genetic diagnosis. The causes of death included renal insufficiency (1), cerebral edema (*n* = 1) and pneumonia (*n* = 1). The cause of death was not known in five cases.

### Rare genetic subtypes (*n* = 9 patients)

#### *EIF2AK3 gene* (*n* = 3 patients, 2 previously reported at presentation^19^)

Recessive pathogenic *EIF2AK3* variants causing Wolcott–Rallison syndrome were detected in three unrelated individuals. Patient 26 was born to distantly related parents and had multiple inpatient admissions due to cytolysis syndrome before dying aged 26 months due to acute renal insufficiency and systemic multiple organ failure. A second patient (Patient 27) was diagnosed with diabetes at the age of 13 weeks and died at 3 months due to cerebral edema. Patient 25 was lost to follow‐up at the age of 4 years. None of the patients had been diagnosed with skeletal dysplasia, one of the cardinal features of Wolcott–Rallison syndrome; however, skeletal dysplasia is often one of the latest features of the syndrome to be diagnosed and, therefore, the lack of skeletal findings at such early age does not exclude the possibility that skeletal dysplasia would have developed later on.

#### *INSR gene* (*n* = 1 patient)

Patient 35 was diagnosed with diabetes at the age of 1 month and was also affected with central hypothyroidism, muscle weakness, developmental delay, malabsorption, anaemia, hyperbilirubinemia, hirsutism, lipoatrophy and facial dysmorphism. Donohue syndrome was suspected and confirmed at 8 weeks, when two heterozygous *INSR* stop‐gain variants, p.(Tyr94Ter) and p.(Arg1020Ter), were detected. While the p.(Tyr94Ter) was confirmed to be maternally inherited, the p.(Arg1020Ter) was not detected in either parent's leukocyte DNA sample. Since non‐paternity was excluded by microsatellite analysis, it is likely that the *INSR* p.(Arg1020Ter) variant had arisen *de novo*. At presentation, the boy received a very high insulin dose (on average 0.15 U/kg/h). Insulin treatment was stopped after 3 weeks, following complaints of fasting hypoglycemia, and subsequently whole‐day hypoglycemias. Hypoglycemic episodes (lowest blood glucose level [BGL] was 1.1 mmol/L) had no clinical symptoms and were compensated with diet and oral glucose. Until 12 weeks of age, the proband periodically presented spontaneous increased BGL up to 19.6 mmol/L with spontaneous dropping to 1.1 mmol/L. BGL was controlled with correction of feeding only. He was first admitted to the ECU due to pneumonia at 8 weeks. He was admitted again at 16 weeks due to refusal of feeding, failure to thrive, shortness of breath and swelling. The patient died at 4 months and 13 days due to systemic multiple organ failure.

#### *PDX1 gene* (*n* = 1 patient)

Patient 36 was diagnosed with diabetes at 17 days and was found to be homozygous for a *PDX1* start loss variant (c.1A > G, p.?). She was a Caucasian female born from a consanguineous marriage (parents are first cousins, the proband's elder sibling died on the 10th day of life, cause unknown). The proband was born 34–35 weeks gestation with a birth weight of 1250 g. She was admitted to the hospital at 5 months due to jaundice, cytolysis syndrome and elevated bilirubin levels; however, she did not have the typical gastrointestinal *PDX1* symptoms (pancreatitis and/or malabsorption). MRI and cholecystocholangiography showed intrahepatic biliary tract hypoplasia and hepato‐lienal syndrome. Her condition initially improved but 1 month after hospital admission, it suddenly worsened due to uncorrected hyperglycemia, cytolysis syndrome, hyperthermic syndrome and acute urinary retention. She was transferred to the ECU where death occurred due to sepsis and multiple organ failure at 6 months and 13 days.

#### *GLIS3 gene* (*n* = 1 patient previously described at presentation^19^)

Patient 32 presented with diabetic ketoacidosis at the age of 1 week. She also had congenital hypothyroidism, polycystic kidneys, and patent foramen ovale. Genetic testing identified compound heterozygous *GLIS3* deleterious variants, p.(Pro444fs)/p.(His647Arg). She is currently 13 years old and has been treated with continuous subcutaneous insulin infusion. She also receives L‐thyroxine (2.8 mcg/kg) due to hypothyroidism. Surgery for a patent foramen ovale was performed at the age of 8 years.

#### *RFX6 gene* (*n* = 1 patient)

Patient 37 was born at 39 weeks gestation with a birth weight of 2800 g and was found to be compound heterozygote for two *RFX6* variants, p.(Met409Thr) and p.(Ser517Arg). There is no known family history of diabetes; both non‐diabetic parents are heterozygous for one of the *RFX6* variants. The proband was diagnosed prenatally with duodenal atresia. After birth, she was found to have congenital malformation of the small intestine, intestinal obstruction, duodenal atresia, annular pancreas, and anaemia. On the seventh day of life, she underwent surgery (diamond‐shaped duodenojejunal anastomosis side to side). Diabetes was diagnosed at 2 days (BGL–20 mmol/L) and insulin treatment was started for 4 weeks in a very small dose (0.1 U/kg/day). The diabetes remitted at 1 month but relapsed when she was 2 months old, when she was found to have low C‐peptide (0.8 ng/ml, normal range 1.1–4.4). Additional examination showed that the proband has exocrine pancreatic insufficiency (fecal elastase 21.9 mcg/g, normal range > 200mcg) requiring enzyme supplementation therapy with Creon. The rectosigmoscopy revealed an erosive‐hemorrhagic proctosigmoiditis. MRI and cholecystocholangiography at 8 months showed duodenal atresia with prestenotic dilatation of the descending part; hypoplasia of the pancreas, minimal Wirsungectasia; agenesis of the gallbladder; moderate ectasia of intra‐ and extrahepatic bile ducts; dystopia of the transverse colon, and atypical location of the dome of the cecum. At the age of 8 months, the child has severe failure to thrive. On a background of constant treatment with ursodeoxycholic acid, she has normal liver function tests but elevated alkaline phosphatase (862 U/L [normal value <460]).

#### *FOXP3* *gene* (*n* = 1 patient)

Patient 33 was born from a second pregnancy on the background of retrochorial hematoma. Polyhydramnios, signs of intrauterine infection and fetal distress were recorded at 32 weeks of gestation. The male proband was born at 40–41 weeks gestation with a birth weight of 3880 g and an Apgar score of 6/7 points. Since birth, the child presented with severe respiratory disorders, with nasal constant positive airway pressure needed from the seventh day of life. At 5 days, the child was transferred to the Regional Children's Clinical Hospital to the neonatal intensive care unit. The child's condition on admission was extremely severe due to diaphragmatic hernia, respiratory failure and suppressed reflexes. On the 16th day of life, a right thoracotomy was performed to correct the right diaphragmatic hernia. After surgery, the child was on mechanical ventilation. An attempt to switch to spontaneous breathing at 23 days was unsuccessful due to respiratory failure. On the 30th day of life, he was diagnosed with diabetes (BGL up to 23.8 mmol/L), and insulin therapy was started. Glycemic control was challenging and satisfactory compensation of diabetes could not be achieved. At 32 days, his C‐peptide was 0.21 ng/ml (normal value 0.9–7.1). General blood tests, biochemical analysis and urine tests did not show significant changes. A diffuse mucopurulent endobronchitis was detected by a bronchoscopy. The child died aged 90 days. Three months after his death, genetic testing identified a pathogenic, maternally inherited hemizygous *FOXP3* variant, p.(Arg347His).

#### *LRBA gene* (*n* = 1 patient)

The proband (Patient 34) was born at 38 weeks gestation with a weight of 3150 g. He was the second child of unrelated healthy parents (an elder sibling was unaffected, a second and third pregnancy had previously terminated with their miscarriage). He was first admitted to the infectious disease department of a district hospital at the age of 5 months with a suspicion of acute intestinal infection. He was treated with antibacterial therapy and parenteral rehydration without significant effect. Two mg of dexamethasone was administered parenterally four times (a total of 8 mg) per day. The child was referred to the NICU where infusion therapy and antibiotics were continued. Further investigations revealed hyperglycemia and decreased C‐peptide levels (<0.05 ng/ml, normal value 0.81–3.85). Insulin therapy was started. The child's condition initially improved, and the boy was discharged in a satisfactory condition to continue outpatient treatment with insulin. Genetic testing was performed in 2014, but no pathogenic variants were identified in the 20 genes known to cause neonatal diabetes at that time. He was admitted again at the age of 9 months, with a preliminary diagnosis of acute gastroenterocolitis, disseminated intravascular coagulation syndrome (DICS), intestinal toxicosis and exicosis (grade 2). Due to further deterioration of his condition, with suspicion of ulcerative necrotic enterocolitis, ileal gangrene, serous peritonitis and intestinal obstruction, an urgent diagnostic laparoscopy was performed, which was extended to direct laparotomy. Given the significant pathological changes in the intestine due to necrotic ulcerative colitis, mesenteric thrombosis, intestinal necrosis, and serous hemorrhagic peritonitis, resection of 40 cm of the ileum and ileostomy were performed. Subsequently, the patient's condition did not improve, and he died of cardiac arrest. The post‐mortem found an abdominal form of nodular periarteritis, complicated by total hemorrhagic gangrene of the small and large intestine, diffuse serous‐hemorrhagic peritonitis as well as focal hemorrhages in the lungs. Four years later, following the identification of *LRBA* biallelic variants as a cause of neonatal/early‐onset diabetes,^16^ further testing was performed and a homozygous frameshift variant in the *LRBA* gene p.(Glu946Ter) was identified.

### Neonatal and early‐onset diabetes due to the common genetic causes (*n* = 24 patients, 18 previously reported at presentation^19^)

Activating pathogenic variants in *ABCC8* or *KCNJ11* were identified in 14 patients from 13 unrelated families diagnosed before 6 months (8 with *KCNJ11* and 5 with *ABCC8*), and one individual diagnosed between 6 and 9 months (who was heterozygous for a *KCNJ11* pathogenic variant). Four individuals with *ABCC8* variants and one with a heterozygous *KCNJ11* variant had TNDM with remission between 1 and 12 months after presentation. The patient with *KCNJ11*‐TNDM (Patient 8 in Table 2) was diagnosed with juvenile rheumatoid arthritis at 8 years and received insulin temporarily during treatment with glucocorticoids. The heterozygous pathogenic *KCNJ11* and *ABCC8* variants had arisen *de novo* in 9 cases (2/5 *ABCC8* and 7/9 *KCNJ11*). One patient (Patient 12 in Table 2) was compound heterozygous for the *ABCC8* variants.^19^ The remaining four cases had inherited the pathogenic variant from their mothers, two of whom (both harboring *KCNJ11* variants) were diagnosed with diabetes at 3 months of age and had been treated with insulin until the genetic diagnosis in their children (aged 29 and 35), (see Table 2). Transfer to sulfonylurea therapy was successful in all probands with activating *KCNJ11* and *ABCC8* variants in our cohort^19^ including the affected mothers.

Three patients with *ABCC8* pathogenic variants had atypical clinical features at follow‐up. Patient 12 who was compound heterozygote for the *ABCC8* variants p.(Val324Met)/p.(Arg1394Leu) had severe neurological features which did not improve when sulfonylurea (SU) treatment was started when he was 6 years old. At the age of 8, he presented with growth failure (height − 4.5 SD) and inguinal cryptorchidism. Additional examinations detected hypopituitarism with low IGF‐1 level (81 ng/ml, normal range 95–460). Treatment with chorionic gonadotropin showed a temporary effect. An orchidopexy was performed when he was 11. Treatment with growth hormone was started at 13 and has not led to worsening of the glycemic control or increasing of SU dose. At the age of 13, the patient still has severe generalized hypotonia and is unable to sit, hold his head upright, walk or talk. Two siblings heterozygous for a *de novo* *ABCC8* p.(Ile49Phe) variant (Patients 10 and 11 in Table 2) had severe developmental delay, epilepsy, and neonatal diabetes (DEND) syndrome. The proband was diagnosed with diabetes at 3 months which remitted at 1 year and relapsed at 2 years. His sister developed convulsions and hypoglycemic coma at 5 months, and at 6 months was diagnosed with diabetes and received insulin for a few days before being transferred to SU treatment. Both siblings had been treated with a low SU dose with excellent glycemic control (HbA_1c_ was stable <7%) until their death. The proband died aged 9 years, 1 day after admission to the ECU because of hyperthermia, cytolysis syndrome, jaundice and systemic multiple organ failure of unknown origin. His 5‐year‐old sister died of pneumonia (she also had severe rickets and curvature of the chest).

The second most common genetic cause of neonatal/early‐onset diabetes in our cohort were heterozygous pathogenic variants in the *INS* gene (*n* = 6 patients, three diagnosed before 6 months). In one family, the pathogenic *INS* p.(Gly32Ser) variant was also detected in the patient's mother and maternal grandmother who were diagnosed with ‘type 1 diabetes’ at 3 years. The disease presentation was atypical in the proband who had neonatal hypoglycemia on the 3rd day of life (glucose level was 2.1 mmol/L), and despite developing diabetes at 5 months, did not start insulin treatment until 2 years of age. We observed a similar presentation in another individual with the same *INS* p.(Gly32Ser) variant who did not need insulin treatment between 2 and 8 months of age.^19^ Thus, two patients with heterozygous *INS* variants in our cohort had remission of diabetes.

Transient neonatal diabetes caused by 6q24 paternal uniparental disomy was identified in 4 patients (3 previously reported^19^). In two cases the diabetes relapsed at 9 and 10 years (HbA_1c_–8.0% and 8.5%, respectively); both are currently treated with diet only. The other two cases are currently aged 2 and 8 years and are still in the remission phase.

Heterozygous *GCK* variants were identified in 4 individuals (Patients 28–31 in Table 2, one previously reported^19^, ^22^). Two of them were diagnosed before the age of 6 months. In all 4 cases, BGL was checked in the asymptomatic infants as their mothers had been diagnosed with gestational diabetes which had not required insulin treatment. Despite the early presentation, all patients underwent genetic testing after 1 year of age, and their fasting hyperglycemia was between 6.1 and 6.9 mmol/L. Patient 30 had one hypoglycemic episode 2 hours after birth. In family 29, the mother and proband's brother, who were diagnosed with diabetes at 30 and 18 years, respectively, were also heterozygous for the pathogenic *GCK* variant, confirming a diagnosis of *GCK*‐MODY.

### Neonatal diabetes diagnosed before 6 months without a confirmed genetic diagnosis (*n* = 5)

No likely causative variant was identified in five patients diagnosed before 6 months of age (Table 3). Patient 38 was diagnosed with impaired glucose tolerance (IGT) at 5 months and 3 weeks and was asymptomatic. She had moderately elevated BGL, low C‐peptide 0.4 ng/ml (normal range 0.81–3.85), and negative pancreatic autoantibodies. Analysis of all the known monogenic diabetes genes did not identify a likely cause; however, she was noted to be heterozygous for a *HNF1A* p.(Thr537Arg) variant of uncertain significance. Further investigations are needed to determine whether the variant is linked to the phenotype or if it is a benign polymorphism. Patient 39 likely had hyperglycemia of prematurity since he was born at 26.5 weeks and the diabetes remitted when he was 4.5 months of age. The remaining 3 unsolved neonatal diabetes patients all had a high T1D GRS suggesting that they are likely to have very early‐onset type 1 diabetes.

**TABLE 3:** Patients with neonatal diabetes diagnosed before 6 months without a pathogenic variant identified

	Clinical diagnosis	Age at diagnosis	Genetic variants being followed up	T1D‐GRS (centile of T1D controls)	HLA	Family history of DM
38	IGT	5 months	Heterozygous *HNF1A* c.1610C > G, p.(Thr537Arg), variant of uncertain significance	NA	NA	No
39	TNDM (hyperglycemia of prematurity)	2 months	None	38th	DR3/X	No
40	PNDM	5 months	None	>95th	DR3/DR3	No
41	PNDM	5 months	None	70th	DR3/DR4	No
42	PNDM	2 months	None	94th	DR3/DR3	Father, T1D at 23 y.o.

## 4 DISCUSSION

In this study we assessed the aetiologies and clinical features of neonatal and early‐onset diabetes occurring within the first 9 months of life in 66 children from Ukraine. Our data show a high proportion of rare genetic causes within our cohort (24.4% of solved cases), including autosomal recessive aetiologies, and a high mortality rate (21.6% of individuals with a confirmed genetic diagnosis). To our knowledge, this is the first study assessing mortality in a large neonatal and early‐onset diabetes cohort in a European country.

Over the last decade, the identification of novel, rarer causes of neonatal/early‐onset diabetes and the availability of comprehensive testing has allowed us to increase our diagnostic yield with almost all our cases with diabetes diagnosed before 6 months having a likely explanation for their early presentation (86.1% having a monogenic cause identified, 8.4% having early‐onset type 1 diabetes, 2.8% having hyperglycemia of prematurity). Testing in individuals diagnosed between 6 and 9 months who were antibody negative identified a genetic cause in 20% of cases, highlighting the importance of considering genetic testing also in this disease group where T1D is much more common.

Our comprehensive testing approach has allowed us to identify cases with rare genetic aetiologies and cases with atypical presentation. Despite Ukraine being a country with a low rate of consanguineous unions, autosomal recessive causes of neonatal/early‐onset diabetes were identified in eight families. In two of these families the parents were known to be related, with compound heterozygous variants identified in the remaining six cases. Our results show the important contribution of autosomal recessive causes of neonatal/early‐onset diabetes, even in a non‐consanguineous setting.

An early genetic diagnosis has important implications for the patients, allowing targeted management of their diabetes. An example of this in our cohort was the early detection of individuals heterozygous for *GCK* pathogenic variants, who are often misdiagnosed. We identified four children with heterozygous *GCK* variants, consistent with them having fasting hyperglycemia from birth (*GCK*‐MODY). *GCK*‐MODY is more commonly diagnosed in adults where hyperglycemia is incidentally picked up; however, in some cases it can be detected in the neonatal period,^19^, ^22^ most commonly due to control of BGL in children by their affected mothers. A genetic diagnosis of *GCK*‐MODY is important in these patients to avoid unnecessary treatment and monitoring and to inform management of pregnancy in affected mothers.

Clinical follow‐up in our cohort highlighted atypical presentation and clinical course of diabetes in some families. Three of our patients (Patient 11 with a heterozygous *ABCC8* variant, patient 16 with a maternally inherited *INS* variant, and patient 30 with a heterozygous *GCK* variant) presented with hypoglycemia in the neonatal period (patient 11 presented with hypoglycemic coma). Co‐existence of hypoglycemias and hyperglycemias has been reported in patients with loss‐of‐function *ABCC8* hyperinsulinism,^23^ resulting from dysregulation of insulin secretion in patients with diffuse *ABCC8* hyperinsulinism.^24^ We can hypothesize that similar dysregulation of insulin secretion may occur also in patients with gain‐of‐function mutations in the KATP channel genes; however, such a presentation in those with pathogenic *INS* and *GCK* gene variants, to the best of our knowledge, is novel. While in our cases we cannot exclude completely the possibility of transitional hypoglycemia, this atypical presentation requires further investigation. Furthermore, we observed remission of diabetes in two patients with heterozygous pathogenic *INS* variants (in one case for more than 1 year). This is unusual as dominant *INS* variants most commonly cause permanent diabetes.

Some of the additional extra‐pancreatic features identified in our cohort were also atypical, for example the presence of hypopituitarism in patient 12 who is compound heterozygous for two *ABCC8* variants. This case also had severe neurological features which did not improve with sulphonylurea treatment, this could be due to delayed diagnosis and treatment with sulphonylurea.^25^, ^26^, ^27^, ^28^ Furthermore, patient 36, who was homozygous for a *PDX1* start‐loss variant, did not have symptoms of pancreatitis and/or malabsorption, but had intrahepatic biliary tract hypoplasia and died in infancy. This is different from the phenotype of isolated neonatal diabetes with or without exocrine insufficiency described in previous reports of individuals with recessive *PDX1* pathogenic variants.^29^, ^30^ Patient 33, who was hemizygous for a pathogenic *FOXP3* variant, also had an atypical clinical course for IPEX syndrome (Immune dysregulation, Polyendocrinopathy, Enteropathy, X‐linked), and did not have any of the classical features of this condition^31^ other than diabetes, although it is possible that these would have developed if the child had survived. For all patients in our cohort, we only performed genetic testing for the known genetic causes of neonatal/early‐onset diabetes, we, therefore, cannot exclude the presence of other genetic variants, which may contribute to the extra‐pancreatic features observed.

For some of the individuals with the rarer genetic subtypes, the extra‐pancreatic features were consistent with the genetic diagnosis. For example, Mitchell‐Riley syndrome caused by biallelic *RFX6* pathogenic variants is characterized by neonatal/early‐onset diabetes, pancreatic hypoplasia, intestinal atresia, and gallbladder aplasia or hypoplasia. Early mortality has been reported in some patients.^32^ Patient 37 in our cohort had typical features of this syndrome, with the exception of the presence of erosive‐hemorrhagic proctosigmoiditis. Similarly, patient 34 had a classical presentation of *LRBA*‐related disease,^16^ namely diabetes and enteropathy. Timely haematopoietic stem cell transplantation^33^ or use of abatacept and/or glucocorticoid therapy can result in a more favorable prognosis in individuals with *LRBA*‐related disease.

We observed a relatively high mortality rate within our cohort, with eight patients dying between infancy and childhood (21.6% of cases with a confirmed diagnosis). All these patients were diagnosed with diabetes before 6 months of age and had a genetic cause identified. The mortality rate is even higher when only considering patients with rare genetic subtypes (6/9, 66.7%). The higher mortality in this group is likely to be due to many different factors, including the fact that extra‐pancreatic complications are common in these patients and therapeutic options, which improve prognosis are currently available only in those with some monogenic forms of autoimmune diabetes.^16^, ^17^, ^33^

Our study had some limitations. First, ZnT8 antibodies were not routinely tested in individuals diagnosed between 6 and 9 months, which may have negatively affected the pick‐up rate in this age group. Furthermore, since we used tNGS for the known genetic causes of diabetes, we cannot exclude the possibility of additional variants in non‐diabetes genes contributing to the atypical presentation and mortality in some cases.

## 5 CONCLUSIONS

The present study highlights the broad spectrum of genetic heterogeneity and clinical presentations of neonatal and early‐onset diabetes in Ukraine, where rare autosomal and X‐linked recessive genetic subtypes, atypical findings and high mortality rate were relatively common. Whereas autoimmune monogenic diabetes, Donohue syndrome and Wolcott–Rallison syndrome are known to have a poor prognosis, death in infancy/childhood in patients with *PDX1* and *ABCC8* pathogenic variants is rare. Implementation of early comprehensive genetic testing including targeted next‐generation sequencing can improve clinical management of this genetically heterogeneous disease.

## AUTHOR CONTRIBUTION

Evgenia Globa performed a clinical investigation of patients at the initial stage and follow‐up, was responsible for conception and design of the study, data acquisition, preparation of the manuscript, finding relevant references, and final approval of the manuscript. Nataliya Zelinska performed a clinical investigation of patients at the initial stage and follow‐up; designed the analyses; reviewed and edited the manuscript. Matthew B Johnson, Sarah E. Flanagan and Elisa De Franco performed and interpreted genetic testing; conceptualized and designed the study; and critically reviewed and revised the manuscript. Elisa De Franco is the guarantor and approved the final manuscript as submitted.

## FUNDING INFORMATION

This work is funded in part by a research grant from the Ministry of Health of Ukraine (0120U000217). EDF is a Diabetes UK RD Lawrence fellow. M.B.J. is the recipient of an Exeter Diabetes Centre of Excellence Independent Fellowship funded by Research England's Expanding Excellence in England (E3) fund. S.E.F. is a Wellcome Trust Senior Research Fellow (Grant Number 223187/Z/21/Z). For the purpose of open access, the author has applied a CC BY public copyright licence to any Author Accepted Manuscript version arising from this submission. Genetic testing for neonatal diabetes until March 2020 was funded by a Wellcome Trust Senior Investigator Award to Professors Sian Ellard and Andrew Hattersley.

## CONFLICT OF INTEREST

The authors declare that they have no conflicts of interest.

## ETHICAL APPROVAL

All procedures performed in the studies involving patients were in accordance with the ethical standards of the institution on clinical practice and with the 1964 Helsinki Declaration, as amended. The parents or legal guardians of patients signed informed consent forms in which they agreed to the treatment and all the diagnostic procedures required. The study was approved by the local ethical committee of Ukrainian Scientific and Practical Center of Endocrine Surgery, Transplantation of Endocrine Organs and Tissues of the Ministry of Health of Ukraine, Kyiv, Ukraine (№ 5, 23.12.2019).

## Data Availability

I confirm that my Data Availability Statement (pasted below) complies with the Expects Data Policy. The data that support the findings of this study are openly available in DECIPHER at https://decipher.sanger.ac.uk/.
